# Supplementary material for: Splice-Junction-Based Mapping of Alternative Isoforms in the Human Proteome
Source: Cell Rep. Author manuscript; Available in PMC 2020 Jan 15. (PMC6961840; doi:10.1016/j.celrep.2019.11.026)
Supplement: 2 [file NIHMS1546469-supplement-2.zip › DF1/PXD010154/Esophagus/Esophagus_28_CCS_GSGQLRVQDVEVHLEDQMVLVHTTLPSQEVQALLEGTGR.pdf]

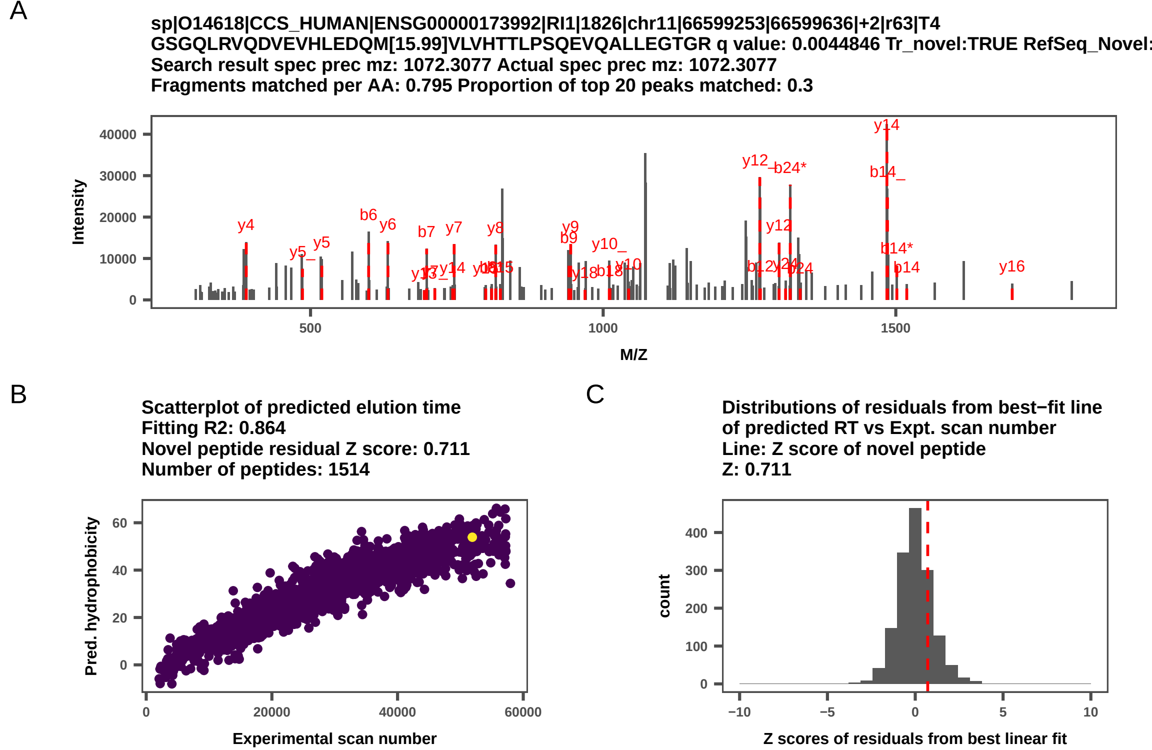
A
sp|O14618|CCS_HUMAN|ENSG00000173992|RI1|1826|chr11|66599253|66599636|+2|r63|T4
GSGQLRVQDVEVHLEDQM[15.99]VLVHTTLPSQEVQALLEGTGR q value: 0.0044846 Tr_novel:TRUE RefSeq_Novel:
Search result spec prec mz: 1072.3077 Actual spec prec mz: 1072.3077
Fragments matched per AA: 0.795 Proportion of top 20 peaks matched: 0.3
Intensity
M/Z
y4
y5_
y5
b6
y6
b7
y7
y8
y9
b9
y10_
y10
y12_
b24*
y12
y14
b14_
b14*
b14
y16
B
Scatterplot of predicted elution time
Fitting R2: 0.864
Novel peptide residual Z score: 0.711
Number of peptides: 1514
Pred. hydrophobicity
Experimental scan number
C
Distributions of residuals from best−fit line
of predicted RT vs Expt. scan number
Line: Z score of novel peptide
Z: 0.711
count
Z scores of residuals from best linear fit
